# Blockade of the renin-angiotensin system suppresses hydroxyl radical production in the rat striatum during carbon monoxide poisoning

**DOI:** 10.1038/s41598-020-59377-6

**Published:** 2020-02-13

**Authors:** Shuichi Hara, Masamune Kobayashi, Fumi Kuriiwa, Hajime Mizukami, Toshiji Mukai

**Affiliations:** 1 Department of Forensic Medicine, Tokyo Medical University, 6-1-1 Shinjuku, Shinjuku-ku, Tokyo 160-8402 Japan; 2 Department of Legal Medicine, Kanazawa Medical University, 1-1 Daigaku, Uchinada-cho, Kahoku-gun Ishikawa, 920-0293 Japan; 3 Department of Legal Medicine, St. Marianna University School of Medicine, 2-16-1 Sugao, Miyamae-ku, Kawasaki City, Kanagawa 216-8511 Japan

**Keywords:** Cell death in the nervous system, Neurodegeneration, Neurotoxicity syndromes

## Abstract

Oxidative stress has been suggested to play a role in brain damage during carbon monoxide (CO) poisoning. Severe poisoning induced by CO at 3000 ppm, but not 1000 ppm, enhances hydroxyl radical (**˙**OH) production in the rat striatum, which might be mediated by NADPH oxidase (NOX) activation associated with Ras-related C3 botulinum toxin substrate (Rac) via cAMP signaling pathway activation. CO-induced **˙**OH production was suppressed by antagonists of angiotensin II (AngII) type 1 receptor (AT1R) and type 2 receptor (AT2R) but not an antagonist of the Mas receptor. Suppression by an AT1R antagonist was unrelated to peroxisome proliferator-activated receptor γ. Angiotensin-converting enzyme inhibitors also suppressed CO-induced **˙**OH production. Intrastriatal AngII at high concentrations enhanced **˙**OH production. However, the enhancement of **˙**OH production was resistant to inhibitors selective for NOX and Rac and to AT1R and AT2R antagonists. This indicates a different mechanism for **˙**OH production induced by AngII than for that induced by CO poisoning. AT1R and AT2R antagonists had no significant effects on CO-induced cAMP production or **˙**OH production induced by forskolin, which stimulates cAMP production. These findings suggest that the renin-angiotensin system might be involved in CO-induced **˙**OH production in a manner independent of cAMP signaling pathways.

## Introduction

Carbon monoxide (CO) is widely recognized as a poisonous gas that binds to hemoglobin in place of oxygen in the blood, forming carboxyhemoglobin (COHb), which hinders oxygen transport to tissues and results in systemic hypoxia. The brain requires substantial amounts of oxygen to function and is therefore vulnerable to CO poisoning. Patients with CO poisoning frequently develop neuropsychiatric symptoms, including parkinsonism and amnesia, and damage to various brain regions^1–3^. Experimental animal models of CO poisoning exhibit similar neuropsychiatric symptoms with brain damage^1,4,5^. Although autopsies have indicated similarities between brain damage by CO poisoning and that by hypoxia unrelated to CO^1^, accumulated evidence suggests that hypoxia due to COHb formation is not the sole mechanism of brain damage in CO poisoning but that multiple mechanisms, in addition to hypoxia, contribute to eventual brain damage^2,3^. Oxidative stress is a key factor that plays various roles in these mechanisms^2,3^.

Oxidative stress in terms of the production of hydroxyl radicals (**˙**OHs), the most cytotoxic reactive oxygen species (ROS), is more profoundly enhanced by CO poisoning than by comparable hypoxic hypoxia in the rat striatum^6^ and hippocampus^7^. Sun *et al*.^5^ demonstrated that brain damage is attenuated by the administration of hydrogen (H_2_), which is a strong scavenger of **˙**OH with a weak ability to scavenge peroxynitrite with no effect on superoxide (O_2_^−^), hydrogen peroxide (H_2_O_2_) or nitric oxide^8^, in a rat model of CO poisoning. It is of interest that **˙**OH production in the rat striatum is stimulated during severe CO poisoning (>70% COHb), but not during less severe poisoning (approximately 50% COHb)^6^. In addition, **˙**OH production parallels cAMP production^9^. CO-induced cAMP production is susceptible to suramin and NF-157, antagonists of the purine P2Y_11_ receptor^9^, which is not expressed in rats^10^. NF-157 attenuates CO-induced **˙**OH production as well^11^. Moreover, the intrastriatal administration of forskolin, an enhancer of cAMP production through the activation of adenylyl cyclase, stimulates **˙**OH production which is susceptible to diphenyleneiodonium (DPI), a nonselective inhibitor of NADPH oxidase (NOX)^11^. The enhancement of **˙**OH production by activators of cAMP signaling pathways is also susceptible to DPI in human granulocytes^12^. Several studies have suggested that ROS production stimulated by CO is at least in part mediated by xanthine oxidase (XO), while NOX is inhibited by CO^3^. We found that CO poisoning decreased the protein levels of several NOX isoforms, including dual oxidases, and Ras-related C3 botulinum toxin substrate (Rac) 1^13^, which is required for the activation of NOX isoforms, such as NOX1 and NOX2^14^, in the rat striatum. Unexpectedly, however, CO-induced **˙**OH production in the rat striatum was strongly suppressed by two different NOX inhibitors, DPI and 4-(2-aminoethyl) benzenesulfonylfluoride (AEBSF; much more selective than DPI for NOX), but not the XO inhibitor, allopurinol^11^. In addition, EHT1864, a Rac inhibitor, strongly suppressed CO-induced **˙**OH production as strongly as DPI^13^. It is likely that CO-induced **˙**OH production is enhanced by the activation of Rac-dependent NOX isoforms mediated through cAMP signaling pathways activated by cAMP production via P2Y_11_-like purinoreceptors. There are conflicting reports, however, that PPADS (pyridoxalphosphate-6-azophenyl-2′,4′-disulfonic acid), a nonselective purine P2X/Y receptor inhibitor, strongly suppresses CO-induced **˙**OH production^11^ without affecting CO-induced cAMP production^9^ and that the intrastriatal administration of ATP, an intrinsic agonist of purine P2X/Y receptors, enhances **˙**OH production, an effect that is susceptible to PPADS, but resistant to NF-157^11^. In addition, it has been hypothesized that CO-induced **˙**OH production might result from an imbalance between stimulation and suppression of **˙**OH production by the activation of the exchange protein directly activated by cAMP (Epac) and protein kinase A (PKA) in cAMP signaling pathways, respectively^15^. Thus, CO-induced **˙**OH production is likely to be mediated through complex mechanisms.

It has been documented that the brain and peripheral organs possess their own renin-angiotensin system (RAS)^16^, which participates in various physiological functions^16,17^. Angiotensin II (AngII) is a major player in the RAS^16,17^ and enhances ROS production by activating NOX via AngII type 1 receptor (AT1R)^18–21^, thus contributing to the development of various brain disorders, including stroke, traumatic brain injury and neurodegenerative diseases, as well as hypertension^18,19,21–23^. Another AngII receptor, AngII type 2 receptor (AT2R)^17^, which has various functions, including modulating neuronal apoptosis and axonal regeneration^23,24^ and antagonizing the effect of AT1R^25^. Although AT1R and AT2R are considered to play a negative regulatory role and no role in cAMP production, respectively^17^, several *in vitro* studies have shown that AngII enhances cAMP production, an effect that is susceptible to AT1R and AT2R antagonists^26–28^. In addition, stimulation of the Mas receptor with angiotensin^1–7^ (Ang^1–7^), which is an AngII metabolite with counterregulatory functions against AngII, results in the enhancement of cAMP production in several cultured cell lines^29^.

Studies have suggested that interfering with the RAS by blocking AT1R with its antagonists^18,19,21–23^ or inhibiting AngII production with angiotensin converting enzyme (ACE) inhibitors^16,30^ may be neuroprotective against various brain insults. If CO-induced **˙**OH production is susceptible to ACE inhibitors and AT1R antagonists, which are widely used for the treatment of hypertension and its complications with high safety, then the RAS may be a novel therapeutic target for brain damage due to CO poisoning. In the present study, we explored the role of the RAS in CO-induced **˙**OH production in the rat striatum.

## Results

### Effects of the modification of the AngII system on CO-induced ˙OH production

**Figure 1:**
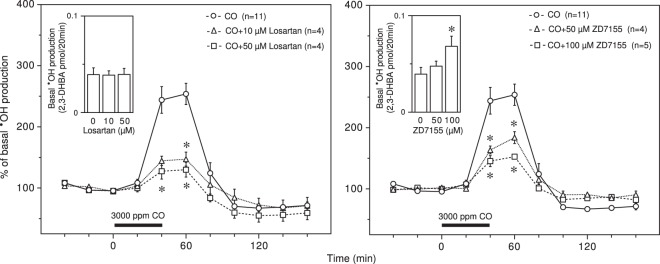
Effects of AT1R antagonists on CO-induced **˙**OH production. Losartan (10 and 50 μM) and ZD7155 (50 and 100 μM) were dissolved in the perfusing medium and administered throughout the experimental period. The inserted graphs depict basal **˙**OH production in terms of 2,3-DHBA formation. Each column or symbol with a vertical bar indicates the mean ± SEM. The horizontal bars indicate exposure of 3000 ppm CO for 40 min. *Significant difference (p < 0.05) from each AT1R antagonist at 0 μM or CO alone by one-way ANOVA followed by Dunnett’s test.

**Figure 2:**
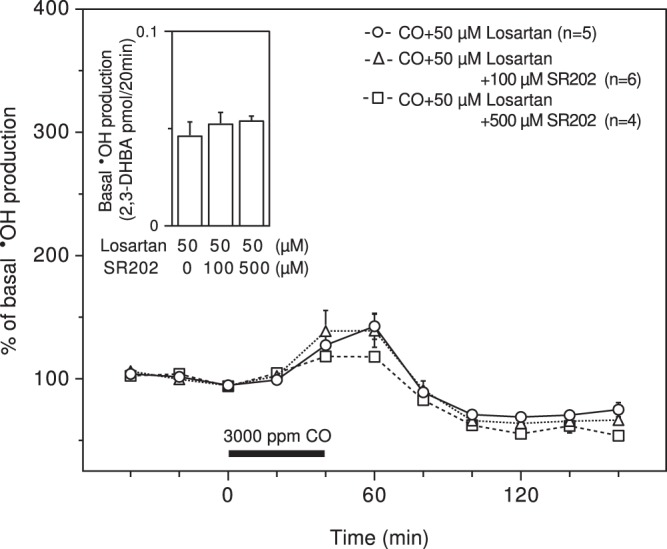
Effect of a PPARγ antagonist on the suppression of CO-induced **˙**OH production by losartan. Losartan (50 μM) and SR202 (100 and 500 μM) were dissolved in the perfusing medium and administered throughout the experimental period. The inserted graph depicts basal **˙**OH production in terms of 2,3-DHBA formation. Each column or symbol with a vertical bar indicates the mean ± SEM. The horizontal bar indicates exposure to 3000 ppm CO for 40 min. There was no significant difference by one-way ANOVA.

The blockade of AT1R with losartan resulted in strong and significant suppression of CO-induced **˙**OH production in a dose-dependent manner with no effect on basal **˙**OH production (Fig. 1 (left)). Another AT1R antagonist, ZD7155, also significantly suppressed CO-induced **˙**OH production in a dose-dependent manner, while it tended to increase basal **˙**OH production in a dose-dependent manner (Fig. 1 (right)). Losartan has the ability to not only antagonize AT1R but also to activate peroxisome proliferator-activated receptor γ (PPARγ), contributing to the alleviation of hepatic injury following ischemia/reperfusion^31^. The PPARγ antagonist SR202 at 100 μM and 500 μM had no effect on basal **˙**OH production in the presence of losartan or the suppression of CO-induced **˙**OH production by losartan (Fig. 2). The raw data for Figs. 1 and 2 are provided in Supplementary Figs. 1 and 2.

**Figure 3:**
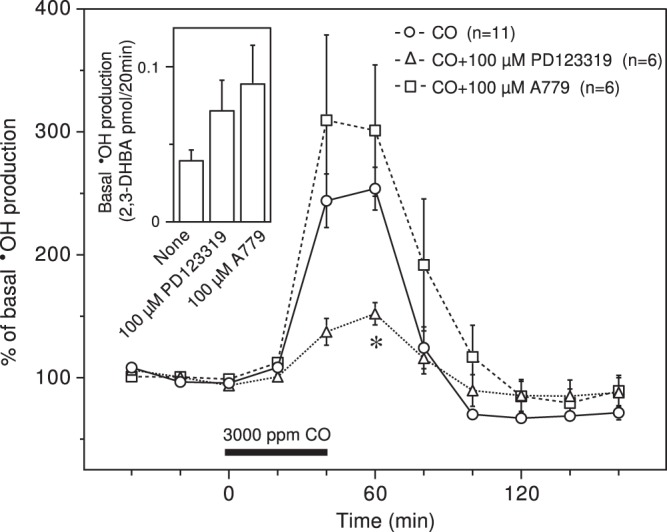
Effects of AT2R and Mas receptor antagonists on CO-induced **˙**OH production. PD123319 (100 μM) and A779 (100 μM) were dissolved in the perfusing medium and administered throughout the experimental period. The inserted graph depicts basal **˙**OH production in terms of 2,3-DHBA formation. Each column or symbol with a vertical bar indicates the mean ± SEM. The horizontal bar indicates exposure to 3000 ppm CO for 40 min. *Significant difference (p < 0.05) from CO alone by one-way ANOVA followed by Dunnett’s test.

The AT2R antagonist PD123319 (100 μM) significantly suppressed CO-induced **˙**OH production and nonsignificantly enhanced basal **˙**OH production (Fig. 3). A779 (100 μM), an antagonist of the Mas receptor that is activated by Ang^1–7^, but not AngII^29^, enhanced basal and CO-induced **˙**OH production, but this effect was not statistically significant (Fig. 3). The raw data for Fig. 3 are provided in Supplementary Fig. 3.

**Figure 4:**
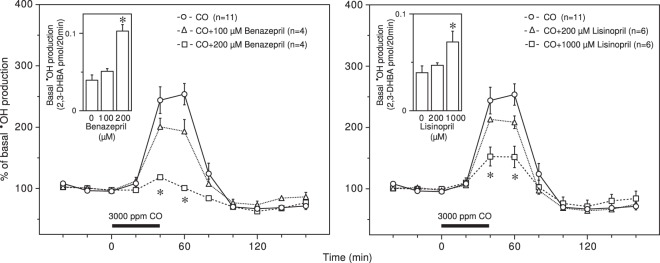
Effect of ACE inhibitors on CO-induced **˙**OH production. Benazepril (100 and 200 μM) and lisinopril (200 μM and 1000 μM) were dissolved in the perfusing medium and administered throughout the experimental period. The inserted graphs depict basal **˙**OH production in terms of 2,3-DHBA formation. Each column or symbol with a vertical bar indicates the mean ± SEM. The horizontal bars indicate exposure to 3000 ppm CO for 40 min. *Significant difference (p < 0.05) from each ACE inhibitor at 0 μM or CO alone by one-way ANOVA followed by Dunnett’s test.

Two ACE inhibitors, benazepril and lisinopril, dose-dependently increased basal **˙**OH production and suppressed CO-induced **˙**OH production (Fig. 4). The effects of these two ACE inhibitors on both types of **˙**OH production were both statistically significant at higher doses (Fig. 4). The enhancement of basal **˙**OH production by the ACE inhibitors might be involved in various endogenous peptides, including substance P, cholecystokinin and amyloid β, since ACE contributes to the degradation of these peptides^32–34^. The raw data for Fig. 4 are provided in Supplementary Fig. 4.

### Effect of 3000 ppm CO on extracellular AngII

The basal AngII level before air exposure was 1.76 ± 0.32 pg/mL (1.69 ± 0.31 fmol/mL), which was close to the minimum detectable concentration of the EIA kit. The AngII level was increased to 133% of the basal level due to air exposure, increased to up to 148% of the basal level due to subsequent CO exposure, and then decreased to 122% of the basal level. However, these changes in AngII did not reach a statistically significant level according to one-way repeated measures ANOVA (p = 0.0623).

### **˙**OH production induced by intrastriatal AngII

**Figure 5:**
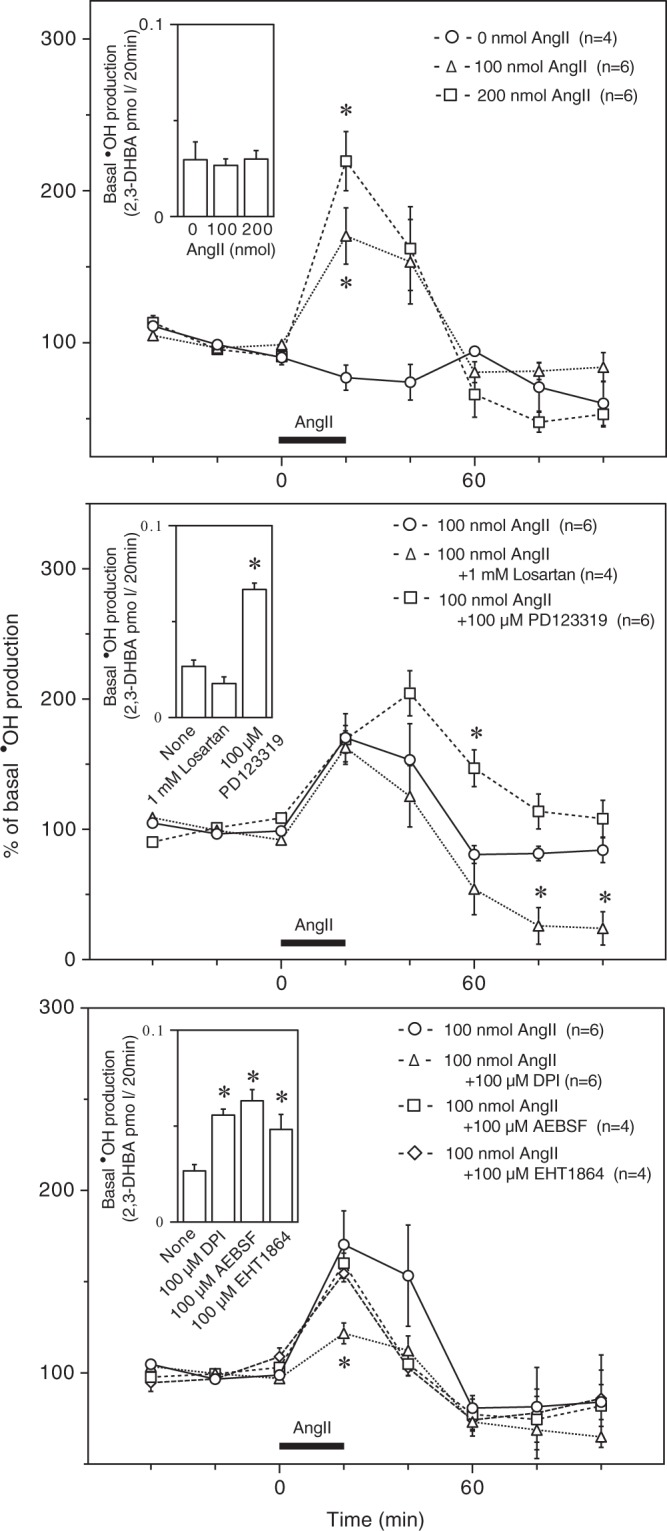
**˙**OH production by intrastriatal administration of AngII (top) and the effects of AT1R and AT2R antagonists (middle) and NOX and Rac inhibitors (bottom) on AngII-induced **˙**OH production. AngII (100 nmol/2 μL or 200 nmol/2 μL) was dissolve in sterilized saline and administered into the striatum at 0.1 μL/min for 20 min. Losartan (1 mM), PD123319 (100 μM), DPI (100 μM), AEBSF (100 μM) and EFT1864 (100 μM) were dissolved in the perfusing medium and administered throughout the experimental period. The inserted graphs depict basal **˙**OH production in terms of 2,3-DHBA formation. Each column or symbol with a vertical bar indicates the mean ± SEM. The horizontal bars indicate administration of AngII. *Significant difference (p < 0.05) from 0 nmol AngII (saline alone) (top) and “None” (neither antagonists nor inhibitors) or 100 nmol AngII alone (middle and bottom) by one-way ANOVA followed by Dunnett’s test.

Presuming that the recovery rate for AngII (1 kDa) in the above microdialysis experiment was 15%, based on the recovery rates of approximately 17% and 13% for interleukin 6 (21 kDa) and amyloid-ß^1–40^ (4 kDa), respectively, we estimated the extracellular AngII concentration to be approximately 11 fmol/mL by *in vitro* microdialysis at a flow rate of 1 µL/min using a probe with a polyethylene membrane^35^. When AngII was directly administered to the striatum, however, much greater amounts of AngII were required to stimulate **˙**OH production in this region, as shown in Fig. 5 (top); intrastriatal AngII at 100 nmol and 200 nmol significantly enhanced **˙**OH production in a dose-dependent manner. **˙**OH production induced by 200 nmol AngII was comparable with that induced by 3000 ppm CO. AngII was rather difficult to dissolve in saline at 200 nmol/2 μL, and the resultant AngII solution was viscous. The following experiments were performed using AngII at 100 nmol/2 μL. Losartan, even at 1 mM, had no effect on basal or AngII-induced **˙**OH production, although it significantly suppressed **˙**OH production at subsequent points in the experiment (Fig. 5 (middle)). PD123319 at 100 μM, which significantly enhanced basal **˙**OH production, did not influence the enhancement of **˙**OH production by AngII administration but delayed the restoration of **˙**OH production enhanced by AngII (Fig. 5 (middle)). Figure 5 (bottom) shows the effects of 100 μM DPI (a nonselective NOX inhibitor), 100 μM AEBSF (a NOX inhibitor that is much more selective than DPI) and 100 μM EHT1864 (an inhibitor of Rac that is required for the activation of some isoforms of NOX, such as NOX1 and NOX2^14^) on AngII-induced **˙**OH production. All three inhibitors significantly enhanced basal **˙**OH production (Fig. 5 (bottom)), although the mechanism of the enhancement is unclear. Neither AEBSF nor EHT1864 had any effect on AngII-induced **˙**OH production, while DPI significantly suppressed AngII-induced **˙**OH production (Fig. 5 (bottom)). The raw data for Fig. 5 are provided in Supplementary Fig. 5.

### Effects of losartan and PD123319 on CO-induced cAMP production and the forskolin-ws-induced **˙**OH production

**Figure 6:**
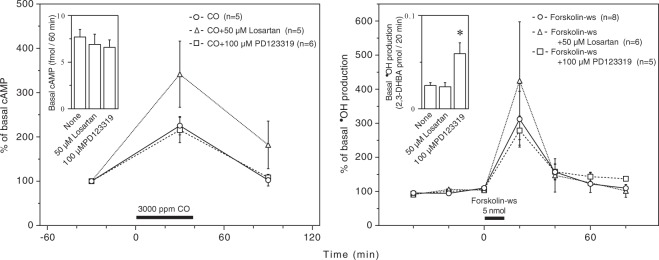
Effects of AT1R and AT2R antagonists on CO-induced cAMP production (left) and forskolin-ws-induced **˙**OH production (right). Losartan (50 μM) and PD123319 (100 μM) were dissolved in the perfusing medium and administered throughout the experimental period. Forskolin-ws (5 nmol/μL) was dissolve in sterilized saline and administered into the striatum at 0.1 µL/min for 10 min. The inserted graphs depict basal cAMP production (left) and basal **˙**OH production in terms of 2,3-DHBA formation (right). Each column or symbol with a vertical bar indicates the mean ± SEM. The horizontal bars indicate 40-min exposure to 3000 ppm CO (left) or administration of 5 nmol forskolin-ws (right). *Significant difference (p < 0.05) from “None” (no antagonists) by one-way ANOVA followed by Dunnett’s test.

As shown in Fig. 6, 3000 ppm CO enhanced cAMP production in the striatum, as previously described^9^, and the intrastriatal administration of 5 nmol forskolin-ws, an enhancer of cAMP production, stimulated **˙**OH production to a level that was comparable to that induced by 3000 ppm CO (Fig. 1). Neither 50 μM losartan nor 100 μM PD123319 influenced basal cAMP production, although the latter significantly enhanced basal **˙**OH production (Fig. 6). Under these conditions, losartan nonsignificantly enhanced both CO-induced cAMP production and forskolin-ws-induced **˙**OH production, while PD123319 had no effects on either of cAMP production or forskolin-ws-induced **˙**OH production (Fig. 6). In the forskolin-ws plus losartan-treated group, **˙**OH production was varied among the individual rats. This variation might be partly due to the dual contradictory mechanisms of **˙**OH production via cAMP signaling pathways^15^ because it is likely that forskolin-ws-induced cAMP production was enhanced by losartan, resulting in the simultaneous activation of both of the mechanisms. The raw data for Fig. 6 are provided in Supplementary Fig. 6.

## Discussion

The present study demonstrated that the enhancement of **˙**OH production was strongly suppressed by AT1R antagonists (i.e., losartan and ZD1755) and ACE inhibitors (i.e., benazepril and lisinopril) in the rat striatum during severe CO poisoning induced by 3000 ppm CO. The PPARγ antagonist (i.e., SR202) had no effect on the suppression of CO-induced **˙**OH production by losartan. The AT2R antagonist PD123319, but not the Mas receptor antagonist (i.e., A779), also suppressed CO-induced **˙**OH production. Losartan and PD123319 had little or no effect on CO-induced cAMP production, which is involved in **˙**OH production via NOX activation^11,13,15^. In addition, administration of exogenous AngII only at nonphysiological concentrations enhanced **˙**OH production, and this enhancement was resistant to losartan, PD123319, a NOX inhibitor (i.e., AEBSF but not DPI), and a Rac inhibitor (i.e., EHT1864).

Suppression of CO-induced **˙**OH production by ACE inhibitors and AT1R blockers strongly suggest that severe CO poisoning induced by 3000 ppm CO may stimulate the RAS in the striatum, accelerating AngII production, which activates AT1R, thus resulting in the enhancement of **˙**OH production. Several AT1R antagonists, including losartan^31^, have the ability to activate PPARγ, accounting for the mechanism of their neuroprotective effects^21,22^. It is unlikely, however, that PPARγ contributes to the suppression of CO-induced **˙**OH production by AT1R blockers, since SR202 had no effect on the suppression of CO-induced **˙**OH production by losartan. The significant suppression of CO-induced **˙**OH production by PD123319 suggests that CO-induced **˙**OH production might be mediated by AT2R in addition to AT1R. This finding contrasts the findings of various studies that indicate the antagonistic functions of AT2R on AT1R^25^. Kilic *et al*.^36^ demonstrated the protective effect of losartan and PD123319 on ischemia-reperfusion injury associated with oxidative stress in isolated rat hearts. In addition, Khanmoradi and Nasimi^37^ suggested that both AT1R and AT2R might share the function of regulating the firing rates of neurons in the paraventricular nucleus, which are involved in the cardiovascular response. As shown in Fig. 3, the enhancement of basal **˙**OH production by PD123319 was not statistically significant but was significant in Figs. 5 and 6. Therefore, AT2R might suppress steady-state **˙**OH production. ZD1755 might act as an AT2R antagonist at higher doses and enhance basal **˙**OH production. AT2R may interact with the Mas receptor that is activated by Ang^1–7^ but not AngII^29^. Although A779 enhanced basal and CO-induced **˙**OH production, this enhancement was not statistically significant. The Mas receptor likely plays a minor role in CO-induced **˙**OH production, while further studies on the role of the Mas receptor in basal **˙**OH production are needed.

We determined the changes in extracellular AngII in the striatum during CO poisoning by means of EIA in combination with brain microdialysis, since it was likely that extracellular AT-II, which acts at AT-II receptors^17^, played a major role in CO-induced ˙OH production. It appeared that CO exposure slightly increased extracellular AngII levels above the level of the small increase induced by exposure to air alone. These changes in the extracellular AngII levels occurred in a range close to the minimum detectable concentration of the EIA, which indicates that further studies should use an EIA with much higher sensitivity to AngII, which is not currently available. Therefore, we examined the effect of the direct administration of exogenous AngII into the striatum on **˙**OH production. **˙**OH production was significantly enhanced by exogenous AngII at doses of 100 nmol and 200 nmol, which were substantially greater than both the extracellular AngII level estimated from the present microdialysis experiments and the doses sufficient to elicit significant cardiovascular responses^37,38^. AngII-induced **˙**OH production was resistant to losartan even at 1 mM, which was 100-fold higher than the concentration sufficient to significantly suppress CO-induced **˙**OH production. PD123319 did not suppress AngII-induced **˙**OH production but seemingly prolonged it. Although AngII-induced **˙**OH production was susceptible to DPI, a nonselective NOX inhibitor, it was resistant to AEBSF, a NOX inhibitor that is more selective than DPI. In addition, AngII-induced **˙**OH production was also resistant to EHT1864. These findings suggest that **˙**OH production may be stimulated by exogenous AngII at nonphysiological concentrations and that AngII-induced **˙**OH production may not be mediated by either AT1R or NOX, while the role of AT2R in AngII-induced **˙**OH production is unclear. Thus, it is unlikely that AngII-induced **˙**OH production shares a mechanism with CO-induced **˙**OH production.

We suggest that the enhancement of **˙**OH production in the rat striatum by severe CO poisoning is due to activation of Rac-dependent NOX isoforms^11,13^ mediated by complex cAMP signaling pathways^15^, which are stimulated by cAMP production via P2Y_11_-like purine receptors^9^. *In vitro* studies using several rodent cell lines have shown that AngII stimulates cAMP production, which is susceptible to losartan and PD123319^26–28^; however, AT1R is coupled to G proteins, which negatively regulate cAMP production, and AT2R has no role in cAMP production^17^. The present study demonstrated that losartan nonsignificantly enhanced CO-induced cAMP production and had no effect on basal cAMP production and that PD123319 affected neither basal nor CO-induced cAMP production. Interestingly, the effects of losartan and PD123319 on CO-induced cAMP production, except for the enhancement of basal **˙**OH production by PD123319, resembled those of forskolin-ws-induced **˙**OH production, which was susceptible to DPI^11^. These observations suggest that AT1R and AT2R antagonists may suppress CO-induced **˙**OH production with little or no influence on cAMP signaling pathways, which lead to NOX activation.

Collectively, the present results suggest that severe CO poisoning might stimulate the RAS in the brain, thereby promoting AngII synthesis, which in turn activates AT1R and AT2R and leads to enhanced **˙**OH production in the rat striatum. In contrast to our previous studies on the mechanism of CO-induced **˙**OH production^13^, this cascade is independent of the cAMP signaling pathways associated with NOX activation, although studies have suggested that AngII activates NOX mediated through AT1R^18–20^. It is likely that the RAS stimulates CO-induced **˙**OH production in cooperation with other systems. Based on the strong suppression of CO-induced **˙**OH production by AT1R blockers in the present study as well as NOX inhibitors in the previous study^13^, it is likely that a close interaction between mechanisms dependent on and independent of cAMP signaling pathways exists. In addition, iron and ascorbate in the brain, which accelerate **˙**OH production^39^, might participate in the interaction^40^.

## Conclusions

The present study demonstrates that interfering with the RAS with ACE inhibitors and AT1R and AT2R antagonists but not the Mas receptor antagonist results in the suppression of **˙**OH production in the striatum of rats with severe CO poisoning. Since it is likely that the suppression of CO-induced **˙**OH production is mediated by a mechanism other than Rac-dependent NOX activation via the cAMP signaling pathway, further studies on the role of the RAS in CO-induced **˙**OH production are needed. However, the present results may indicate novel therapeutic strategies for patients with CO poisoning using ACE and/or AT1R antagonists, which have been clinically and safely used worldwide for the treatment of hypertension and its complications for years.

## Material and Methods

### Animals

Male Sprague-Dawley rats (Charles River Laboratories Japan; Kanagawa, Japan) were kept in an animal facility with controlled temperature (22–24 °C) and a 12-h/12-h light/dark cycle (lights on between 08:00 and 20:00) with free access to food and water for at least one week before all of the experiments.

All experiments were performed in accordance with the Fundamental Guidelines for the Proper Conduct of Animal Experiments and Related Activities in Academic Research Institutions under the jurisdiction of the Ministry of Education, Culture, Sports, Science and Technology, Japan. The experimental protocol for this study was approved by the Institutional Animal Care and Use Committee (IACUC) of Tokyo Medical University.

### Stereotaxic surgery

Stereotaxic surgery was performed under isoflurane (Pfizer Japan, Tokyo, Japan) anesthesia^15^. The rats were individually mounted in a stereotaxic headholder that was equipped with a mask (David Kopf; CA, USA) connected to an inhalation apparatus (The 410 Anaesthesia Unit; Univentor; Zejtun, Malta). A guide cannula (AG-8 or MI-AG-8; Eicom; Kyoto, Japan) for the determination of ˙OH or cAMP levels was unilaterally (left) implanted in the striatum at coordinates (0.2 mm AP, 3.0 mm L and 3.5 mm DV) according to the rat brain atlas^41^. For the determination of extracellular AngII levels, PEG-8 guide cannulae (Eicom) were bilaterally implanted at the abovementioned coordinates. The guide cannulae were secured to the calvarium with miniature stainless-steel screws (AN-3; Eicom) and acrylic dental cement (Quick Resin O; Shofu; Kyoto, Japan) and was plugged with a solid dummy cannula (AD-8, MI-AD-8 or PED-8; Eicom). The rats were given at least five days to recover from the surgery. The location of the dialysis probes was verified after each experiment.

### Exposure of rats to CO

The rats (8- or 9-week-old, weighing 300–410 g) were individually placed in a plastic chamber (26.5 cm in diameter, 28.5 cm in height), which was located in a draft chamber. Air alone or air mixed with CO gas (>99.95%; Sumitomo Seika Chemicals; Tokyo, Japan) was introduced into the chamber at a flow rate of 8 L/min. The CO concentration in the chamber was adjusted to 3000 ppm using a gas flow regulator (Koflok; Osaka, Japan) and a CO monitor (CM-525HB; Gastec; Kanagawa, Japan). The exposure of the rats to 3000 ppm CO for 40 min caused CO poisoning with over 70% blood COHb^6^.

### Measurement of **˙**OH production

**˙**OH production was estimated by measuring the extracellular levels of 2,3-dihydroxybenzoic acid (2,3-DHBA) formed through the nonenzymatic hydroxylation of salicylic acid^42^ according to the protocol of Teismann and Ferger^43^ with modifications^44^. A microdialysis probe with a cellulose membrane (3 mm in length and 0.22 mm in diameter) with or without a thin fused-silica needle (0.15 mm in diameter) for drug administration (MI-A-I-8-03 or A-I-8-03, respectively; Eicom) was inserted into the striatum through the guide cannula and perfused with modified Ringer’s solution (147 mM NaCl, 3 mM KCl, 1.3 mM CaCl_2_, and 1 mM MgCl_2_) containing 5 mM sodium salicylate (Nacali Tesque; Kyoto, Japan) at a flow rate of 2 μL/min using a microsyringe pump (ESP-32; Eicom). The dialysate was collected in an autoinjector (EAS-20; Eicom) in 40-μL fractions every 20 min and injected into an inert HPLC system (Eicom) that was equipped with an electrochemical detector (ECD-300; Eicom) consisting of a graphite working electrode at +500 mV vs. an Ag/AgCl reference electrode. Separation was carried out on an Eicompac SC-5ODS column (2.1 × 150 mm) at 25 °C with a mobile phase that consisted of 100 mM sodium phosphate buffer (pH 6.0) containing 13.4 μM EDTA and 2% (v/v) methanol at a flow rate of 230 μL/min.

### Measurement of extracellular cAMP

The intracellular production of cAMP in the brain can be estimated *in vivo* by measuring extracellular cAMP levels using microdialysis^45–47^. Exposing rats to 3000 ppm CO for 40 min profoundly stimulated cAMP production and **˙**OH production in the striatum during the periods of the second half of CO exposure and in the 20 min after the termination of the exposure^6,9^. In the present study, the dialysate samples were collected every 60 min, specifically 60 min before (basal) and after the start of CO exposure and the subsequent 60 min.

An A-I-8-03 microdialysis probe was inserted into the striatum through the guide cannula and was perfused with modified Ringer’s solution (147 mM NaCl, 3 mM KCl, 1.3 mM CaCl_2_, and 1 mM MgCl_2_) at a constant flow rate of 2 μL/min using an ESP-32 pump. The dialysate fractions were collected in polypropylene tubes on ice and then stored at −80 °C until cAMP analysis (within one month). cAMP was assayed using an Amersham cAMP Biotrac Enzymeimmunoassay (EIA) Kit (GE Healthcare, Buckinghamshire, UK).

### Measurement of extracellular AngII

Microdialysis probes with 3 m- long and 0.44 mm-diameter polyethylene membranes (PEP-8-03; Eicom) were inserted into the right and left striata through the guide cannulae and perfused with modified Ringer’s solution (147 mM NaCl, 3 mM KCl, 1.3 mM CaCl_2_, and 1 mM MgCl_2_) containing 0.15% bovine serum albumin (Boehringer-Ingelheim; Ingelheim, Germany) at a constant flow rate of 1 μL/min using an ESP-32 pump. The bilateral dialysate fractions were collected in polypropylene tubes on ice every 60 min, specifically the first 60 min period for the basal level with no gas introduction into the chamber (basal), the second 60 min period with introduction of air alone for 40 min followed by no gas for 20 min, the third 60 min period with introduction of 3000 ppm CO for 40 min followed by no gas for 20 min, and the fourth 60 min period with no gas introduction. The fractions were stored at −80 °C until AngII analysis (within one month). AngII was assayed using a RayBio Human/Mouse/Rat AngII EIA Kit (RayBiotech, Norcross, GA, USA).

### Drugs and their administration

Losartan, lisinopril and AngII were purchased from Wako Pure Chemical (Tokyo, Japan). Benazepril was purchase from Tokyo Chemical Industry (Tokyo, Japan). PD123319, ZD7155, A779, SR202 and EHT1864 were purchased from Tocris Bioscience (Bristol, UK). Diphenylene iodium (DPI) and 4-(2-aminoethyl) benzenesulfonyl fluoride (AEBSF) were purchased from Sigma-Aldrich. A water-soluble derivative of forskolin, 7-deacetyl-7-[O-(N-methylpiperazino)-γ-butyryl]-forskolin (forskolin-ws), was purchased from Calbiochem (San Diego, CA, USA).

Antagonists of AT1R (losartan and ZD7155) and AT2R (PD123319), the Mas receptor (A779) and PPARγ (SR202) and inhibitors of ACE (benazepril and lisinopril), NOX (DPI and AEBSF) and Rac (EHT1864) were dissolved in the perfused solution and administered to the striatum through the probe during the experimental period. Forskolin-ws, which stimulates cAMP production by activating adenylyl cyclase^48^, and AngII were dissolved in sterilized physiological saline (Otsuka Pharmaceutical, Tokyo, Japan) and directly administered into the striatum through the thin needle of the MI-A-I-8-03 probe using an ESP-32 pump. The flow rate was 0.1 μL/min, and the total volume was 1 μL for forskolin-ws and 2 μL for AngII.

### Statistics

**˙**OH production data are expressed as the percentages of the basal level, which was determined by averaging three consecutive dialysate samples in individual rats before CO exposure. AngII and cAMP data are expressed as the percentages of the individual basal levels. Statistical analysis was performed using a one-way analysis of variance (ANOVA) followed by Dunnett’s test for multiple-group comparisons, one-way repeated measures ANOVA, and Student’s *t* test.

## Supplementary information

Supplementary figures and tables.
