# Supplementary material for: Blockade of the renin-angiotensin system suppresses hydroxyl radical production in the rat striatum during carbon monoxide poisoning
Source: Sci Rep. 2020 Feb 13;10:2602. doi: 10.1038/s41598-020-59377-6 (PMC7018774; doi:10.1038/s41598-020-59377-6)
Supplement: Supplementary file 1 — Supplementary figures and tables. [file 41598_2020_59377_MOESM1_ESM.pdf]

Role of the renin-angiotensin system on hydroxyl radical production in the rat striatum during carbon monoxide poisoning

Shuichi Hara 1), Masamune Kobayashi 2), Fumi Kuriiwa 3), Hajime Mizukami 2), and Toshiji Mukai 3)

1) Department of Forensic Medicine, Tokyo Medical University ,Tokyo 160-8402, Japan
2) Department of Legal Medicine, Kanazawa Medical University, Ishikawa 920-0293, Japan
3) Department of Legal Medicine, St. Marianna University School of Medicine, Kanagawa 216-8511, Japan

			2,3-DHBA (pmol/20 min)						2,3-DHBA (pmol/20 min)					
			Losartan						ZD7155					
Drugs	Gas	Time	0 μM (n=11)		10 μM (n=4)		50 μM (n=4)		0 μM (n=11)		50 μM(n=4)		100 μM (n=5)	
in the perfusing solution	explosure	(min)	Mean	se	Mean	se	Mean	se	Mean	se	Mean	se	Mean	se
Losartan		-40	0.04267	0.00775	0.04046	0.00587	0.04291	0.00699	0.04267	0.00775	0.04688	0.00518	0.06692	0.00962
or		-20	0.03854	0.00720	0.03963	0.00451	0.03820	0.00663	0.03854	0.00720	0.04731	0.00500	0.06944	0.01149
ZD7155		0	0.03688	0.00599	0.03627	0.00337	0.03736	0.00567	0.03688	0.00599	0.04884	0.00610	0.06819	0.01032
	3000 ppm	20	0.04040	0.00558	0.04018	0.00582	0.03849	0.00418	0.04040	0.00558	0.04793	0.00571	0.07514	0.01224
	CO	40	0.10018	0.02192	0.05511	0.00371	0.04788	0.00217	0.10018	0.02192	0.07794	0.00924	0.09608	0.01320
		60	0.09990	0.01743	0.05632	0.00555	0.04900	0.00255	0.09990	0.01743	0.08754	0.01085	0.10413	0.01241
		80	0.05834	0.02230	0.04141	0.00920	0.03193	0.00266	0.05834	0.02230	0.05411	0.00512	0.06990	0.01229
		100	0.02777	0.00528	0.03342	0.00821	0.02198	0.00129	0.02777	0.00528	0.04295	0.00527	0.05960	0.01050
		120	0.02636	0.00476	0.02837	0.00604	0.01987	0.00167	0.02636	0.00476	0.04312	0.00527	0.06201	0.01112
		140	0.02690	0.00509	0.02635	0.00628	0.02040	0.00174	0.02690	0.00509	0.04038	0.00408	0.06275	0.01292
		160	0.02749	0.00500	0.02773	0.00628	0.02166	0.00228	0.02749	0.00500	0.04341	0.00652	0.05898	0.01343
		Basal	0.03936	0.00695	0.03879	0.00445	0.03949	0.00634	0.03936	0.00695	0.04768	0.00517	0.06818	0.01047

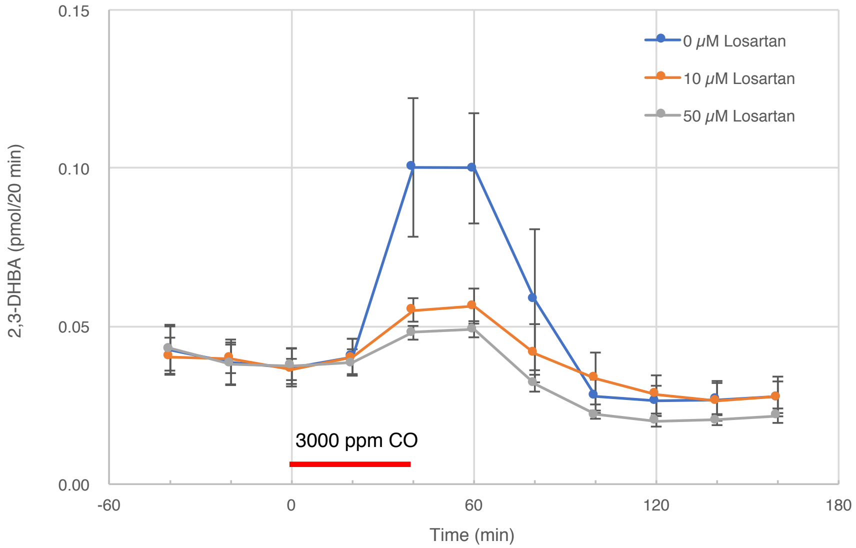

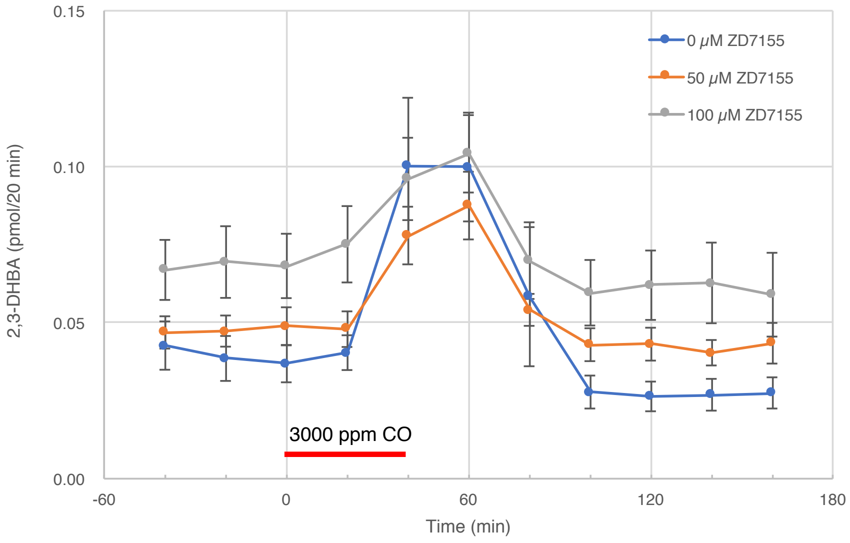

Supplementary Fig. 1. Effects of AT1R antagonists (losartan and ZD7155) on CO-induced ˙OH production in terms of 2,3-DHBA formation. The graphs were produced using the raw values of 2,3-DHBA formation shown in the corresponding tables. Each symbol with a vertical bar indicates the mean ± SEM. The horizontal bars indicate exposure to 3000 ppm CO for 40 min. The AT1R antagonists were dissolved in the perfusing medium and administered throughout the experimental period. % data obtained from simple calculation using the raw values do not depict graphs identical to those in Fig. 1, in which % data were obtained on the basis of the basal 2,3-DHBA levels in individual rats (See Materials and methods).

Drugs in the perfusing solution	Gas explosure	Time (min)	2,3-DHBA (pmol/20 min) SR202 0 μM (n=5) Mean	se	100 μM (n=6) Mean	se	500 μM (n=4) Mean	se
50 μM Losartan		-40	0.04779	0.00734	0.05519	0.00568	0.05510	0.00292
+		-20	0.04678	0.00736	0.05242	0.00680	0.05618	0.00437
SR202		0	0.04395	0.00726	0.04943	0.00591	0.05041	0.00208
	3000 ppm	20	0.04627	0.00811	0.05390	0.00718	0.05626	0.00298
	CO	40	0.06144	0.01412	0.07130	0.00832	0.06333	0.00256
		60	0.06753	0.01501	0.07153	0.00790	0.06329	0.00269
		80	0.04134	0.00728	0.04900	0.00956	0.04430	0.00199
		100	0.03332	0.00650	0.03538	0.00618	0.03355	0.00320
		120	0.03157	0.00465	0.03372	0.00473	0.02955	0.00263
		140	0.03308	0.00607	0.03458	0.00451	0.03290	0.00282
		160	0.03547	0.00731	0.03496	0.00458	0.02882	0.00153
		Basal	0.04617	0.00731	0.05235	0.00608	0.05390	0.00301

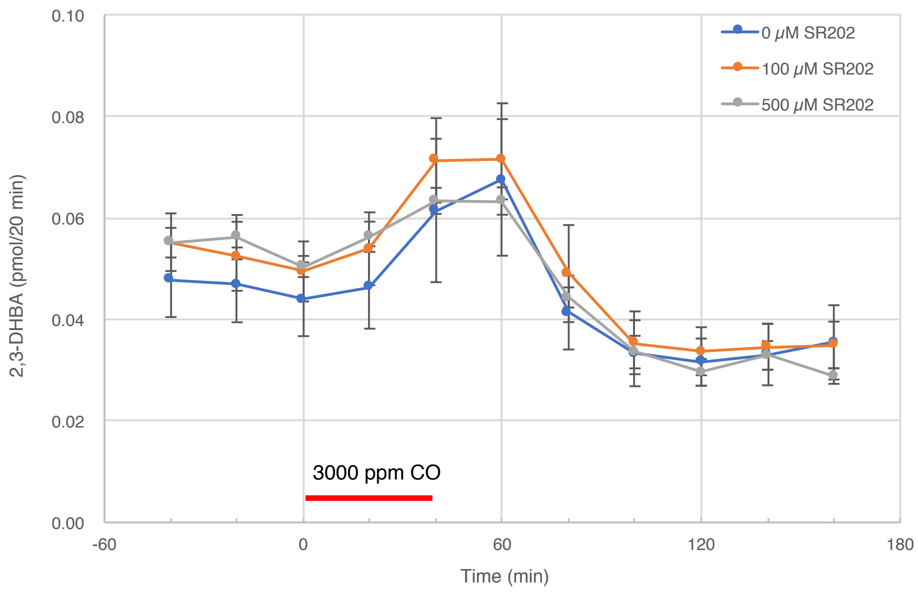

Supplementary Fig. 2. Effect of a PPARγ antagonist (SR202) on the suppression of CO-induced ˙OH production, in terms of 2,3-DHBA formation, by losartan (50 μM). The graph was produced using the raw values of 2,3-DHBA formation shown in the table. Each symbol with a vertical bar indicates the mean ± SEM. The horizontal bar indicates exposure to 3000 ppm CO for 40 min. Losartan and SR202 were dissolved in the perfusing medium and administered throughout the experimental period. % data obtained from simple calculation using the raw values do not depict a graph identical to that in Fig. 2, in which % data were obtained on the basis of the basal 2,3-DHBA levels in individual rats (See Materials and methods).

Drugs in the perfusing solution	Gas explosure	Time (min)	2,3-DHBA (pmol/20 min) No drugs Control (n=11) Mean	se	PD123319 100 μM(n=6) Mean	se	A779 100 μM (n=6) Mean	se
PD123319 or A779		-40	0.04267	0.00775	0.07417	0.01901	0.09058	0.02720
		-20	0.03854	0.00720	0.07186	0.01985	0.09006	0.02584
		0	0.03688	0.00599	0.06858	0.02056	0.08595	0.02207
	3000 ppm CO	20	0.04040	0.00558	0.07336	0.02101	0.10008	0.02823
		40	0.10018	0.02192	0.09562	0.02277	0.34996	0.17890
		60	0.09990	0.01743	0.10418	0.02249	0.31619	0.14842
		80	0.05834	0.02230	0.08291	0.02239	0.22025	0.13030
		100	0.02777	0.00528	0.06667	0.02087	0.11951	0.05922
		120	0.02636	0.00476	0.06373	0.02086	0.06922	0.01785
		140	0.02690	0.00509	0.06421	0.02167	0.06009	0.00935
		160	0.02749	0.00500	0.06585	0.02175	0.06642	0.00913
		Basal	0.03936	0.00695	0.07154	0.01979	0.08886	0.02492

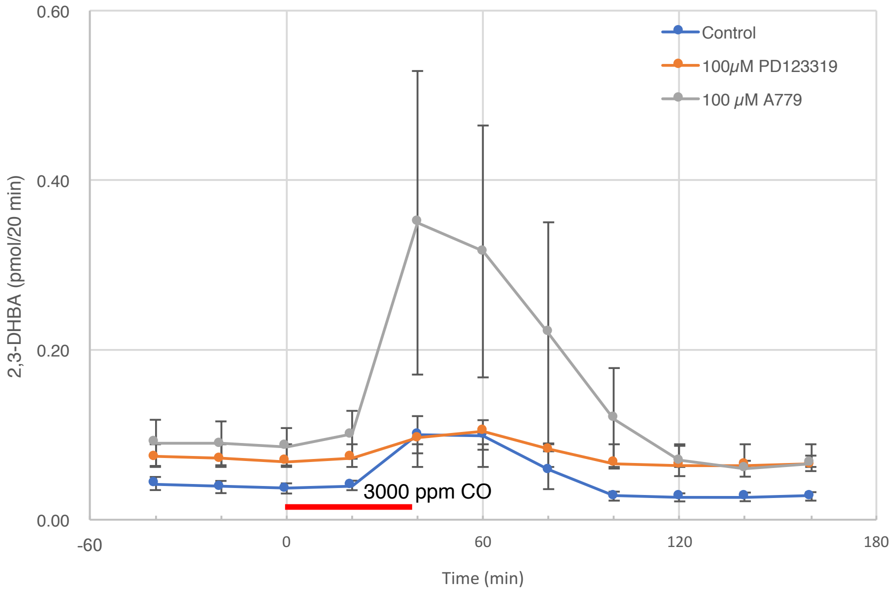

Supplementary Fig. 3. Effects of AT2R and Mas receptor antagonists (PD123319 and A779, respectively) on CO-induced $^{\bullet}$OH production in terms of 2,3-DHBA formation. The graph was produced using the raw values of 2,3-DHBA formation shown in the table. Each symbol with a vertical bar indicates the mean ± SEM. The horizontal bar indicates exposure to 3000 ppm CO for 40 min. The antagonists were dissolved in the perfusing medium and administered throughout the experimental period. % data obtained from simple calculation using the raw values do not depict a graph identical to that in Fig. 3, in which % data were obtained on the basis of the basal 2,3-DHBA levels in individual rats (See Materials and methods).

			2,3-DHBA (pmol/20 min)						2,3-DHBA (pmol/20 min)					
			Benazepril						Lisinopril					
Drugs	Gas	Time	0 μM (n=11)		100 μM (n=4)		200 μM (n=4)		0 μM (n=11)		200 μM (n=6)		1000 μM (n=6)	
in the perfusing solution	explosure	(min)	Mean	se	Mean	se	Mean	se	Mean	se	Mean	se	Mean	se
Benazepril		-40	0.04267	0.00775	0.05240	0.00458	0.10521	0.00867	0.04267	0.00775	0.04740	0.00275	0.06998	0.00912
or		-20	0.03854	0.00720	0.05148	0.00335	0.10342	0.00886	0.03854	0.00720	0.04804	0.00331	0.07068	0.01096
Lisinopril		0	0.03688	0.00599	0.04913	0.00289	0.10010	0.00777	0.03688	0.00599	0.04552	0.00194	0.07232	0.01482
	3000 ppm	20	0.04040	0.00558	0.05677	0.00269	0.10073	0.01118	0.04040	0.00558	0.04960	0.00289	0.08031	0.01872
	CO	40	0.10018	0.02192	0.10346	0.01405	0.12113	0.00879	0.10018	0.02192	0.09996	0.00513	0.11153	0.02829
		60	0.09990	0.01743	0.10012	0.01672	0.10322	0.00774	0.09990	0.01743	0.09831	0.00807	0.11166	0.03027
		80	0.05834	0.02230	0.05543	0.00722	0.08606	0.00657	0.05834	0.02230	0.04592	0.00376	0.07698	0.02164
		100	0.02777	0.00528	0.03968	0.00568	0.07197	0.00682	0.02777	0.00528	0.03242	0.00287	0.05764	0.01813
		120	0.02636	0.00476	0.03800	0.00545	0.06471	0.00687	0.02636	0.00476	0.03040	0.00294	0.05378	0.01705
		140	0.02690	0.00509	0.04309	0.00412	0.06982	0.00726	0.02690	0.00509	0.03150	0.00266	0.06032	0.01816
		160	0.02749	0.00500	0.04420	0.00490	0.07877	0.00799	0.02749	0.00500	0.03512	0.00218	0.06375	0.02030
		Basal	0.03936	0.00695	0.05100	0.00344	0.10291	0.00838	0.03936	0.00695	0.04698	0.00256	0.07099	0.01123

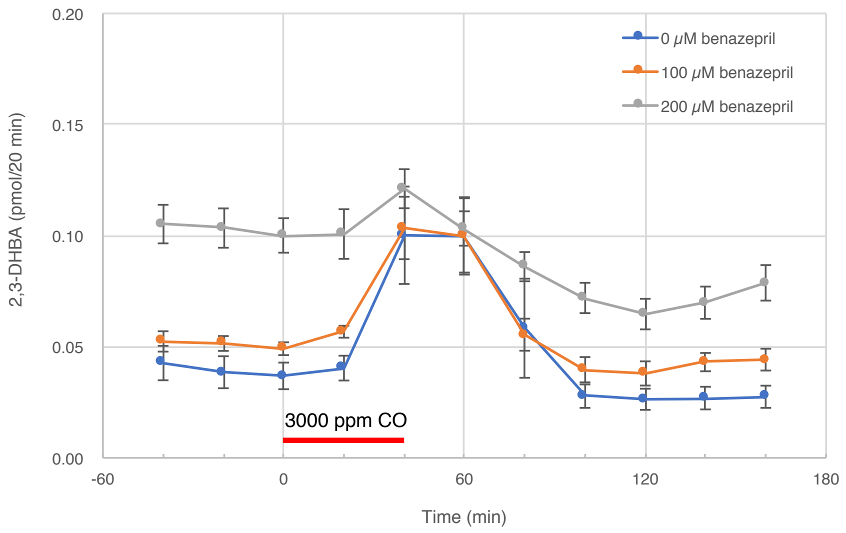

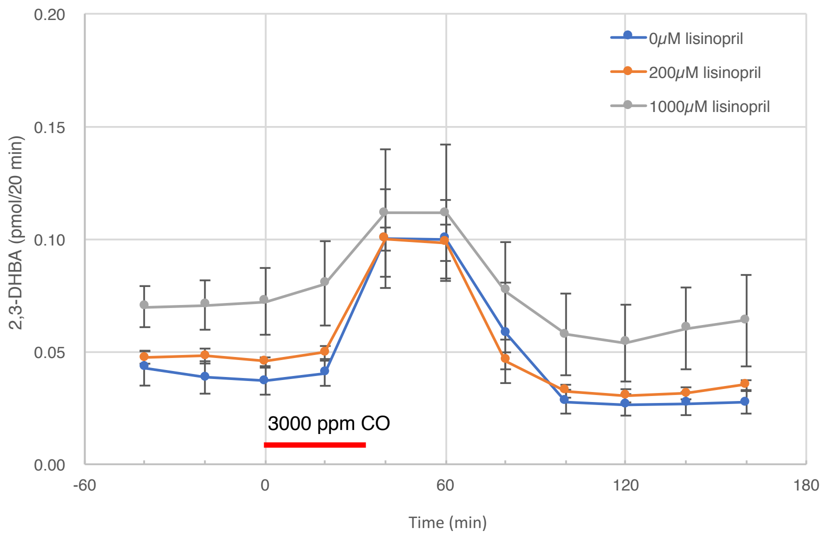

Supplementary Fig. 4. Effect of ACE inhibitors (benazepril and lisinopril) on CO-induced $^{\cdot}$OH production in terms of 2,3-DHBA formation. The graphs were produced using the raw values of 2,3-DHBA formation shown in the corresponding tables. Each symbol with a vertical bar indicates the mean ± SEM. The horizontal bars indicate exposure to 3000 ppm CO for 40 min. The ACE inhibitors were dissolved in the perfusing medium and administered throughout the experimental period.% data obtained from simple calculation using the raw values do not depict graphs identical to those in Fig. 4, in which % data were obtained on the basis of the basal 2,3-DHBA levels in individual rats (See Materials and methods).

Drugs in the perfusing solution	Intrastriatal (2 μL/20 min)	Time (min)	2,3-DHBA (pmol/20 min) AngII 0 nmol (n=4) Mean	se	100 nmol (n=6) Mean	se	200 nmol (n=6) Mean	se
None		-40	0.03113	0.00851	0.02804	0.00353	0.03385	0.00521
		-20	0.02961	0.00937	0.02598	0.00358	0.02867	0.00445
		0	0.02802	0.00998	0.02598	0.00308	0.02717	0.00400
	AngII or Saline	20	0.02475	0.00922	0.04348	0.00454	0.06354	0.00799
		40	0.02455	0.00948	0.03892	0.00708	0.04649	0.00790
		60	0.02773	0.00841	0.02076	0.00203	0.01857	0.00333
		80	0.02337	0.00907	0.02228	0.00206	0.01375	0.00194
		100	0.02021	0.00813	0.02134	0.00277	0.01385	0.00249
		Basal	0.02959	0.00928	0.02667	0.00333	0.02990	0.00451

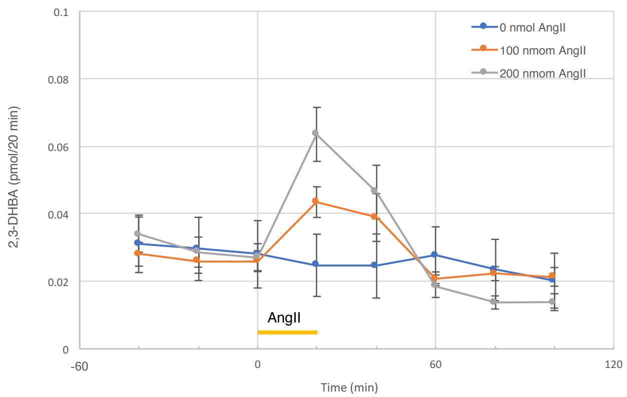

Drugs in the perfusing solution	Intrastriatal (2 μL/20 min)	Time (min)	2,3-DHBA (pmol/20 min) No drugs Control (n=6) Mean	se	Losartan 1 mM (n=4) Mean	se	PD123319 100 μM (n=6) Mean	se
Losartan or PD123319		-40	0.02804	0.00353	0.01933	0.00362	0.06022	0.00374
		-20	0.02598	0.00358	0.01771	0.00358	0.06763	0.00381
		0	0.02598	0.00308	0.01625	0.00316	0.07244	0.00349
	100 nmol AngII	20	0.04348	0.00454	0.02824	0.00509	0.11290	0.00962
		40	0.03892	0.00708	0.02166	0.00543	0.13519	0.01046
		60	0.02076	0.00203	0.01049	0.00455	0.09736	0.00945
		80	0.02228	0.00206	0.00550	0.00311	0.07553	0.00896
		100	0.02134	0.00277	0.00440	0.00331	0.07107	0.00743
		Basal	0.02667	0.00333	0.01776	0.00344	0.06676	0.00330

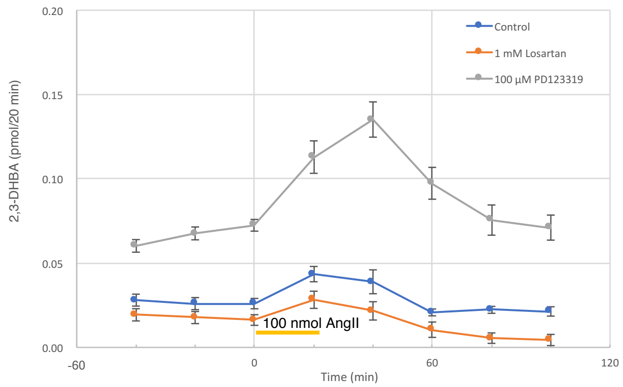

Drugs in the perfusing solution	Intrastriatal (2 μL/20 min)	Time (min)	2,3-DHBA (pmol/20 min) No drugs Control (n=6) Mean	se	DPI 100 μM (n=6) Mean	se	AEBSF 100 μM (n=4) Mean	se	EHT1864 100 μM (n=4) Mean	se
DPI or AEBSF or EHT1864		-40	0.02804	0.00353	0.05774	0.00333	0.04487	0.00579	0.06147	0.00499
		-20	0.02598	0.00358	0.05556	0.00312	0.04640	0.00697	0.06291	0.00617
		0	0.02598	0.00308	0.05395	0.00274	0.05362	0.01096	0.06515	0.00633
	100 nmol AngII	20	0.04348	0.00454	0.06861	0.00660	0.07481	0.01287	0.10175	0.01118
		40	0.03892	0.00708	0.06305	0.00617	0.04960	0.00768	0.06575	0.00617
		60	0.02076	0.00203	0.04038	0.00459	0.03472	0.00368	0.04782	0.00437
		80	0.02228	0.00206	0.03749	0.00298	0.03542	0.01181	0.04437	0.00549
		100	0.02134	0.00277	0.03575	0.00317	0.03962	0.01129	0.04869	0.00678
		Basal	0.02667	0.00333	0.05575	0.00305	0.04829	0.00779	0.06318	0.00583

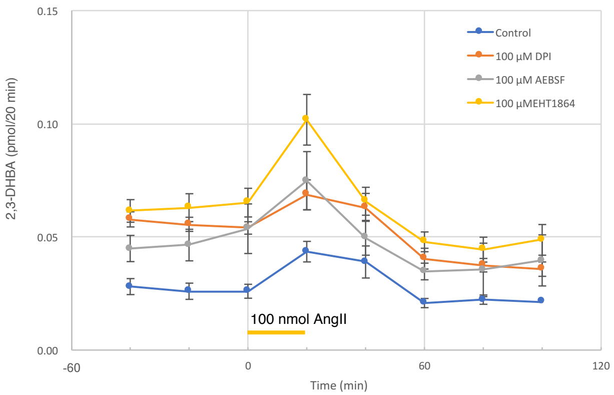

Supplementary Fig. 5. ˙OH production, in terms of 2,3-DHBA formation, by intrastriatal administration of AngII (top) and the effects of AT1R and AT2R antagonists (middle) and NOX and Rac inhibitors (bottom) on AngII-induced ˙OH production. The graphs were produced using the raw values of 2,3-DHBA formation shown in the corresponding tables. Each column or symbol with a vertical bar indicates the mean ± SEM. The horizontal bars indicate administration of AngII dissolved in saline into the striatum at 0.1 μL/min for 20 min. Losartan, PD123319, DPI, AEBSF and EFT1864 were dissolved in the perfusing medium and administered throughout the experimental period. % data obtained from simple calculation using the raw values do not depict graphs identical to those in Fig. 5, in which % data were obtained on the basis of the basal 2,3-DHBA levels in individual rats (See Materials and methods).

			cAMP (fmol/60 min)					
			No drugs		Losartan		PD123319	
Drugs in the perfusing solution	Gas explosure	Time (min)	Control (n=5)		50 μM (n=5)		100 μM (n=6)	
			Mean	se	Mean	se	Mean	se
Losartan		-60 - 0	7.68	0.80	6.86	1.10	6.59	0.80
or	3000 ppm CO (0 - 40 min)	0 - 60	16.75	1.55	23.52	6.47	14.72	2.97
PD123319		60 - 120	7.49	0.78	12.48	4.06	7.18	1.10

			2,3-DHBA (pmol/20 min)					
			No drugs		Losartan		PD123319	
Drugs in the perfusing solution	intrastriatum (1 μL/10 min)	Time (min)	Control (n=8)		50 μM (n=6)		100 μM (n=5)	
			Mean	se	Mean	se	Mean	se
Losartan		-40	0.02423	0.00345	0.02181	0.00487	0.05507	0.01075
or		-20	0.02391	0.00352	0.02509	0.00502	0.05860	0.01109
PD123319		0	0.02661	0.00254	0.02360	0.00405	0.06347	0.01340
	5 nmol Forskolin-ws (0 -10 min)	20	0.07737	0.01914	0.07838	0.02616	0.15252	0.02139
		40	0.03751	0.00507	0.02814	0.00509	0.08846	0.01391
		60	0.02988	0.00357	0.02472	0.00296	0.08388	0.01654
		80	0.02689	0.00307	0.02123	0.00342	0.07889	0.01252
		Basal	0.02491	0.00308	0.02350	0.00453	0.06453	0.01336

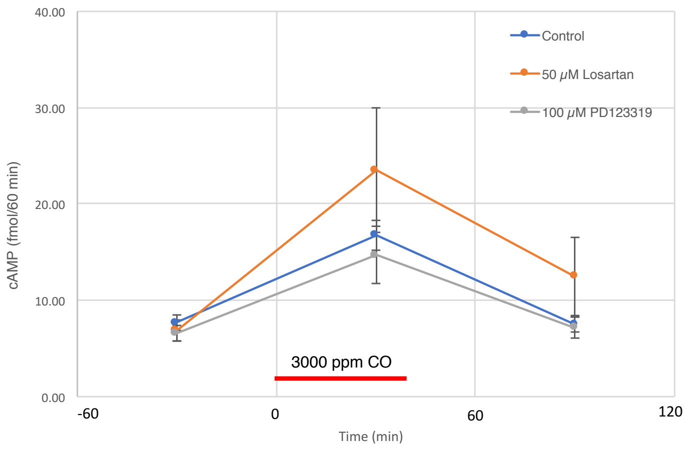

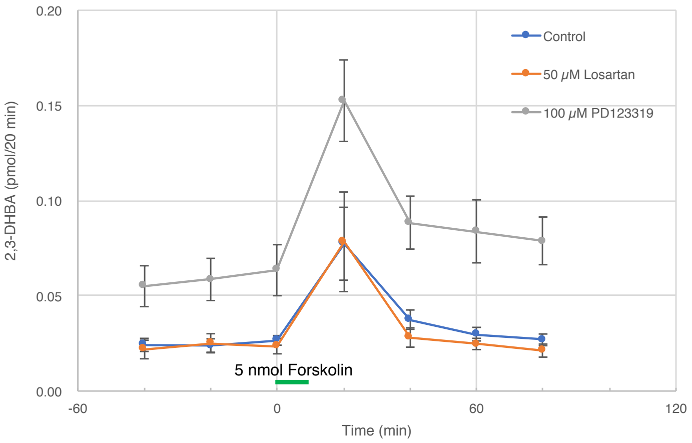

Supplementary Fig. 6. Effects of AT1R and AT2R antagonists on CO-induced cAMP production (left) and forskolin-ws-induced ˙OH production in terms of 2,3-DHBA (right). The graphs were produced using the raw values of cAMP or 2,3-DHBA formation shown in the corresponding tables. Each column or symbol with a vertical bar indicates the mean ± SEM. The horizontal bars indicate 40-min exposure to 3000 ppm CO (left) or administration of 5 nmol forskolin-ws (dissolved in sterilized saline) into the striatum at 0.1 μL/min for 10 min (right). Losartan and PD123319 were dissolved in the perfusing medium and administered throughout the experimental period. % data obtained from simple calculation using the raw values do not depict graphs identical to those in Fig. 6, in which % data were obtained on the basis of the basal 2,3-DHBA levels in individual rats (See Materials and methods).
